# Biological Inoculant of Salt-Tolerant Bacteria for Plant Growth Stimulation under Different Saline Soil Conditions

**DOI:** 10.4014/jmb.2009.09032

**Published:** 2020-12-25

**Authors:** Ru Wang, Chen Wang, Qing Feng, Rey-May Liou, Ying-Feng Lin

**Affiliations:** 1 College of Environmental Science and Engineering, Qilu University of Technology (Shandong Academy of Sciences), Jinan 250353, P.R. China; 2 Department of Research and Development Centre of Ecological Engineering and Technology, Chia Nan University of Pharmacy and Science, Tainan 71710, Taiwan, P.R. China

**Keywords:** Salt-tolerant bacteria, salt stress, plant growth-promoting traits, *Bacillus*, *Brevibacterium frigoritolerans*

## Abstract

Using salt-tolerant bacteria to protect plants from salt stress is a promising microbiological treatment strategy for saline–alkali soil improvement. Here, we conducted research on the growthpromoting effect of *Brevibacterium frigoritolerans* on wheat under salt stress, which has rarely been addressed before. The synergistic effect of *B. frigoritolerans* combined with representative salttolerant bacteria *Bacillus velezensis* and *Bacillus thuringiensis* to promote the development of wheat under salt stress was also further studied. Our approach involved two steps: investigation of the plant growth-promoting traits of each strain at six salt stress levels (0, 2, 4, 6, 8, and 10%); examination of the effects of the strains (single or in combination) inoculated on wheat in different salt stress conditions (0, 50, 100, 200, 300, and 400 mM). The experiment of plant growth-promoting traits indicated that among three strains, *B. frigoritolerans* had the most potential for promoting wheat parameters. In single-strain inoculation, *B. frigoritolerans* showed the best performance of plant growth promotion. Moreover, a pot experiment proved that the plant growth-promoting potential of co-inoculation with three strains on wheat is better than single-strain inoculation under salt stress condition. Up to now, this is the first report suggesting that *B. frigoritolerans* has the potential to promote wheat growth under salt stress, especially combined with *B. velezensis* and *B. thuringiensis*.

## Introduction

Salt tolerant bacteria are salt-associated microorganisms that are known to effectively promote plant growth in saline–alkali soil. For the past few years, a great many salt tolerant bacteria have been proven to promote each part of plant growth. For example, *Pseudomonas* spp. and *Bacillus* spp. can produce auxin, siderophores, and 1-aminocyclopropane-1-carboxylic acid (ACC) deaminase activity in the rhizosphere for promoting plant growth [1-3]. *Bacillus* spp. were frequently isolated from the saline–alkali soil, and most species have shown positive effects on plant growth [4, 5]. *Bacillus* and *Pseudomonas* species reportedly have positive influence on wheat growth [6]. However, treatment of wheat seeds with *Bacillus* has been shown to be more effective than treatment with *Pseudomonas* in plant growth promotion in saline soil. *Brevibacterium frigoritolerans* as a novel organism can effectively degrade 89.81% of the residual phorate in soil [7]. It has also been reported that *B. frigoritolerans* can tolerate 5% NaCl stress, and greatly reduce the accumulation of Se in plants [8]. Nevertheless, there have been fewer studies of *B. frigoritolerans* as a salt-tolerant species capable of alleviating salt stress and promoting plant growth.

Soil salinization has caused a large decrease in total cultivated land and further reduced crop production. Wheat, the largest grain crop in the world, is the main food source for human beings. However, the germination and growth of wheat could be significantly inhibited under salt stress [9]. At present, methods of saline–alkali soil improvement mainly include the establishment of drainage and irrigation systems, deep ploughing, planting of salt-tolerant plants, application of biodegradable organic fertilizers, and the addition of chemicals [10]. However, it is difficult to achieve the expected effect in the management of saline-alkali soil due to the defects of low efficiency, high implementation cost, and unsustainability of these methods. Plant growth-promoting rhizobacteria (PGPR) are well-known plant-associated microorganisms that promote plant growth by various mechanisms such as improving the bioavailability of mineral nutrients for plants [11]. But a major difficulty in applying PGPR technology to saline–alkali soils is that the reproductive speed and activity of microorganisms are inhibited by high salt stress. Therefore, the study of salt-tolerant bacteria that can alleviate salt stress and promote plant growth has gained increasing attention in recent years.

A research survey has shown that the universally representative salt-tolerant microorganisms *Bacillus thuringiensis* and *Bacillus velezensis* can tolerate high concentrations of NaCl. *B. thuringiensis* has effective IAA (indole-3-acetic acid), siderophore, and phosphate-solubilizing ability [1, 2]. Although the ability of *B. velezensis* to promote plant growth is closely related to its IAA and ACC deaminase activities, its siderophore capacity and nitrogen fixation ability have not been detected [12]. Therefore, it is necessary to combine *B. thuringiensis* and *B. velezensis* to form a complementary mechanism, so as to better achieve the goal of plant growth promotion. Interestingly, *Brevibacterium antiquum* strain AY243344 showed positive plant growth-promoting traits including siderophore capacity, phosphate solubilization, and IAA production [13]. In addition, *B. frigoritolerans* strain SMA23 is endowed with plant growth-promoting activities such as siderophore capacity, phosphate solubilization, and IAA production under abiotic stress at different temperatures [14]. *B. frigoritolerans* as a salt-tolerant microorganism has favorable salt tolerance [8, 15], but there have been few studies on its application to promote plant growth under salt stress. Therefore, the plant growth-promoting activities of *B. frigoritolerans* under salt stress needed further study.

In this work, a few species of salt-tolerant bacteria were isolated from saline–alkali soil and their plant growth-promoting characteristics (ACC deaminase activity, IAA production, phosphate solubilization, nitrogen fixation, and siderophore) were evaluated under different levels of salt stress. Finally, the plant growth promotion of these salt-tolerant bacteria was evaluated by pot experiment using wheat.

## Materials and Methods

### Chemicals

IAA and chrome-azurol s (CAS) were purchased from Aladdin, China. 1-aminocyclopropane-1-carboxylic acid (ACC) was purchased from Macklin, China. Anaerobic broth was purchased from Beijing Land Bridge Technology Co., Ltd., China. All the standards used in this study including NaCl, KH_2_PO_4_, glucose, and other media reagents, were purchased from Sinopharm Chemical Reagent Co., Ltd., China. All chemicals used in this study were analytical and biochemical reagents.

### Isolation of Bacteria

To collect soil samples, four different areas with salinity problems were selected in Shandong Province, China. Table 1 shows the saltiness, water content, and volatile solid (VS) results of the collected saline–alkali soil samples that were analyzed. The salinity was measured according to a standard method (2009). In brief, the soil was put in deionized water and shaken for 3 min. The mixed solution was filtered to remove impurities, and then the filtered solution was heated at 105°C. Finally, the residue was weighted to calculate the salinity. The water content and VS were measured by a muffle furnace at 105°C and 600°C, respectively, according to a previous study [16]. Isolation of salt-tolerant bacteria from saline soil was implemented, in which 10 g soil samples were taken respectively from four areas, and added into 100 ml sterile distilled water. All samples were kept on a shaker (120 rpm) at 28°C for 30 min. The soil diluent was spread on anaerobic agar and marine agar, both of which contained 2% NaCl, and the inverted plate culture method was used for incubation at 28°C for 48–72 h.

### Identification of Isolate

The 16s rDNA gene sequence analysis was applied to identify the isolates. Genomic DNA of the isolate was extracted using a DP302 test kit. Two primers of 27F (5'-AGAGTTTGATCMTGGCTCAG-3') and 1492R (5'-TACGGYTACCTTGTTACGACTT-3') [17] were used for PCR amplification. The PCR amplification system contained: 25 μl of 2× PCR Master Mix, 2 μl of 27F, 2 μl of 1492R, 1 μl of genomic DNA, and 20 μl of double distilled water (ddH_2_O). Agarose gel electrophoresis was used to separate DNA and protein. A 1.0% agarose gel was prepared, and the voltage was set to 18 V/cm during electrophoresis. The amplicon of 1.5 kb was purified using the DP214 DNA Purification Extraction Kit (Tiangen Biotech (Beijing) Co., Ltd.). The obtained sequence data of the 16s rDNA were compared with sequences of the GenBank database using the Basic Local Alignment Search Tool (BLAST) program of NCBI.

### Screening for Salt Tolerance

The purified isolates were used to domesticate their salt tolerance. The isolates were inoculated in the broth medium supplemented with various concentrations of 4, 6, 8, and 10% NaCl step-by-step. The sterile anaerobic and marine broth was used as a control. The nutrient broth was incubated on a rotary shaker (120 rpm) at 28°C for 48–72 h.

### Plant Growth-Promoting Activities

**ACC deaminase activity.** The ACC deaminase activity of strains was assayed quantitatively according to a modification of the method from Penrose and Glick [18]. The ability of strain growth on DF (Dworkin and Foster) medium without (NH_4_)_2_SO_4_ and ADF medium supplemented with 2 g/l ACC successively with different salt levels of 0, 2, 4, 6, 8, and 10% NaCl was evaluated. The non-saline medium (0% NaCl) was used as a control for comparison. ACC deaminase activity was assayed by considering the amount of α-ketobutyrate produced when the enzyme ACC deaminase cleaves to α-ketobutyrate and ammonia, by comparing the absorbance at 540 nm of a sample to a standard curve of α-ketobutyrate.

**IAA production.** IAA production of isolates was quantitatively determined based on the method of Gordon and Weber [19]. A 2 ml aliquot of seed culture was cultured in a test tube containing 8 ml nutrient broth supplemented with different concentrations of 0, 2, 4, 6, 8, and 10% NaCl and 100 mg/l tryptophan, and then incubated in a constant temperature (28°C) shaker. After 48 h, 2 ml of bacteria supernatants was mixed with 4 ml Salkowski reagent, and kept in the dark for 30 min. The optical density was then measured at the absorbance of 540 nm. The amount of IAA was determined from the standard curve of IAA.

**Phosphate solubilization.** To determine the phosphate solubilization ability, isolates were inoculated in Pikovskaya’s broth medium [20]. The tricalcium phosphate [Ca_3_(PO_4_)_2_] was the sole source of inorganic phosphate. Quantitative determination of phosphate solubilization ability was based on the method of Fiske and Subbarow [21]. The Pikovskaya’s broth was supplemented with different concentrations of 0, 2, 4, 6, 8, and 10%NaCl, and shaken at 28°C for 7 days. After 7 days, the supernatant was obtained by centrifugation at 8,413 ×*g* for 5 min. The concentration of soluble phosphate in the supernatant was calculated from a standard curve of KH_2_PO_4_.

**Nitrogen fixation.** Ashby agar was used to determine the nitrogen fixation ability of the isolates [22]. The bacterial suspension during logarithmic growth period was centrifuged, and then re-suspended with distilled water. A 6 μl of re-suspension was inoculated on the Ashby agar with different salt levels of 0, 2, 4, 6, 8, and 10% NaCl, contained in 6 mm filter paper that was sterilized in advance, with four inoculation points per dish. After incubation at 30°C for several days, the presence of bacterial growth indicate that the strain has the ability to fix nitrogen.

**Siderophore production.** Siderophore production ability was tested based on the CAS agar analytical method [23]. The bacterial suspension during logarithmic growth period was centrifuged, and then re-suspended with distilled water. Six microliters of re-suspension was inoculated on the CAS agar with different salt concentrations of 0, 2, 4, 6, 8, 10% NaCl, contained in 6 mm filter paper that was sterilized in advance. After incubation at 30°C for several days, the presence of a halo zone around the colony indicated that the strain has the ability to produce siderophore.

### Determination of Ethylene Content

The sterilized wheat seeds were separately soaked in bacterial cultures of *B. velezensis*, *B. thuringiensis*, and *B. frigoritolerans* and the combination of these 3 strains for 2 h. The wheat seeds soaked in the same strain were divided into five bottles of 100 ml, with each containing 200 grains. NaCl in different concentrations (50, 100, 200, 300, 400 mM) was added to each sealed bottle. The wheat seeds soaked in sterile broth served as a control. With the elapse of seven days, 1 ml of gas in the headspace was taken from each bottle and injected into a gas chromatograph (GC-2014C, Shimadzu, Suzhou, China) to measure the ethylene content with a flame ionization detector [24].

### Treatment of Wheat for Assessment of Plant Growth

Based on salt-tolerant bacteria properties, isolates were evaluated for their ability to resist salt stress in plants. Pot experiments were carried out in the growth incubator to evaluate their wheat growth-promoting effects by the inoculation of isolates as single strains or in combination. The method of wet sterilization was adopted to sterilize soil, and wheat seeds were surface-sterilized in the solution containing 75% alcohol and 10% H_2_O_2_, respectively. To impose salt stress, 200 g samples of aliquot sterilized soil were supplemented with 0, 50, 100, 200, 300, and 400 Mm of NaCl solution separately, and after being air dried, were inoculated with isolate. The seeds after sterilization were soaked in bacteria broth for 4 h, and then dried for 1–2 h at room temperature. The treated seeds were put on the plate that was covered with wet filter paper for germination at 25°C. After 3–4 days, seedlings were removed and planted in plastic pots filled with 200 g treated soil, and regularly inoculated. Seedlings sprouted after soaking in sterile broth were used as control. After 28 days, plant parameters such as plant height, root length, and fresh and dry weight, were measured.

### Statistical Analysis

The statistical analysis of the effects of isolates on the growth parameters of plants at different salt stress levels and using different inoculants were analyzed by two-way ANOVA using SPSS version 12.0 (SPSS Inc., USA), and mean comparisons were conducted using a least significant difference (LSD) test (*p*-value = 0.05). Results were presented as an average of the three determinations. Standard error and LSD results were calculated.

## Results and Discussion

### Isolation and Identification of Salt-Tolerant Bacteria

Table 1 shows that no soil sample had a salinity that exceeded 2%. From those soil samples, a total of 8 salt-tolerant microorganisms, denoted as A-1 – A-6 (A refers to anaerobic broth), M-1, and M-2 (M refers to to marine broth), were isolated based on their ambient salinity (2%). Out of the eight isolates, 6 isolates were grown on anaerobic agar in a facultative environment with suitable temperature and pH, while two isolates were grown on marine agar under a natural environment with the same temperature and pH. The experimental strains that were isolated from a soil habitat have unique adaptability in the new soil environment compared with the commercial strains [25].

The nucleotide sequences of each strain were respectively compared with the NCBI GenBank database. Table 2 presents the information on taxonomical identification and GenBank accession numbers of eight isolates. Target strains A-1, A-5, and M-1 were selected for subsequent experiments. As shown in Table 2, A-1 and A-5 are categorized into the genus *Bacillus* [2, 26]. The cells are gram-positive, rod-shaped, facultative anaerobic bacteria of the genus *Bacillus* which occur frequently in chains. M-1 is a gram-positive, short-rod bacterium existent in the genus *Brevibacterium* [27], which is classified as aerobic bacteria.

### Screening for Salt Tolerance

**Fig. 1:**
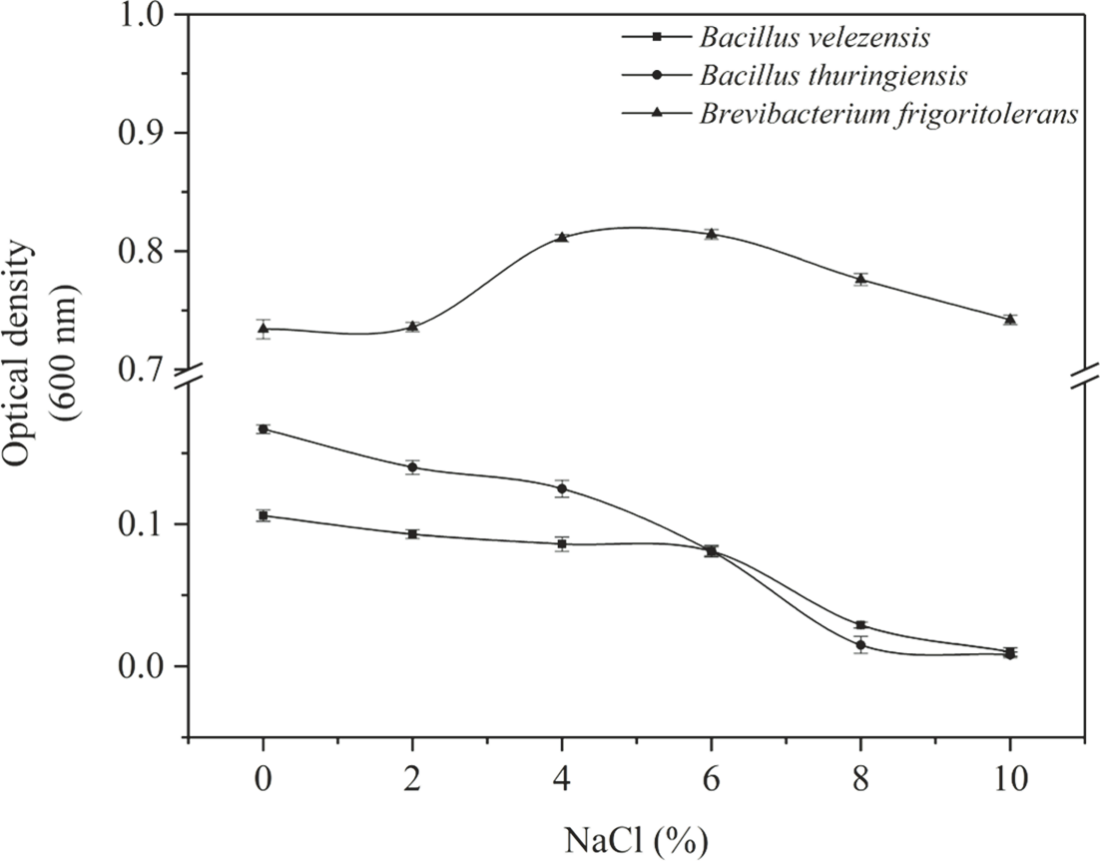
Bacterial growth under different salinity concentrations.

The target salt-tolerant microorganisms were domesticated to tolerate higher concentrations of NaCl, which can play a biological role in higher salt stress. After step-by-step domestication, the three target microorganisms were capable of tolerating up to 10% NaCl concentration. To study the salt requirement of the selected microorganisms, they were cultured at different salinity of 0, 2, 4, 6, 8, and 10% (Fig. 1). *B. frigoritolerans* were grown in saline condition and their optimal growth salinity was around 6%. The isolates of *B. velezensis* and *B. thuringiensis* were cultured in salt concentration ranging from 0–10%, with an optimal growth salinity in the range of 0–5%. As shown in Fig. 1, the isolated strains are capable of growing at a stable OD under the condition of 0-10% NaCl stress. Thus, salinity of 0–10% was adopted to study the role of the three target strains in promoting plant growth.

To monitor bacterial growth, the optical density (OD, λ = 600 nm) of bacteria in broth media was measured using an ultraviolet spectrophotometer [28, 29]. A significant positive correlation was identified between the OD and bacteria concentrations [30]. Previous study has reported the salt-tolerance capacity of *B. velezensis* and *B. thuringiensis* as being up to 10%, but with the increase of salt concentration, the growth activities of the strains decreased greatly [31]. In this study, the growth activity of *B. velezensis* and *B. thuringiensis* decreased with the increase of salt concentration. However, they still showed stable growth activity (Fig. 1). As shown in Fig. 1, the OD_600 nm_ of *B. frigoritolerans* was the highest, suggesting that *B. frigoritolerans* performs best in growth activity among the three strains under the same salt stress. These results show that the three strains could stably grow under salt stress. Thus, these isolates that were adapted to adverse conditions of survival, and could support their hosts in tolerating salt stress environments.

### Plant Growth-Promoting Traits of Salt-Tolerant Bacteria

**Fig. 2:**
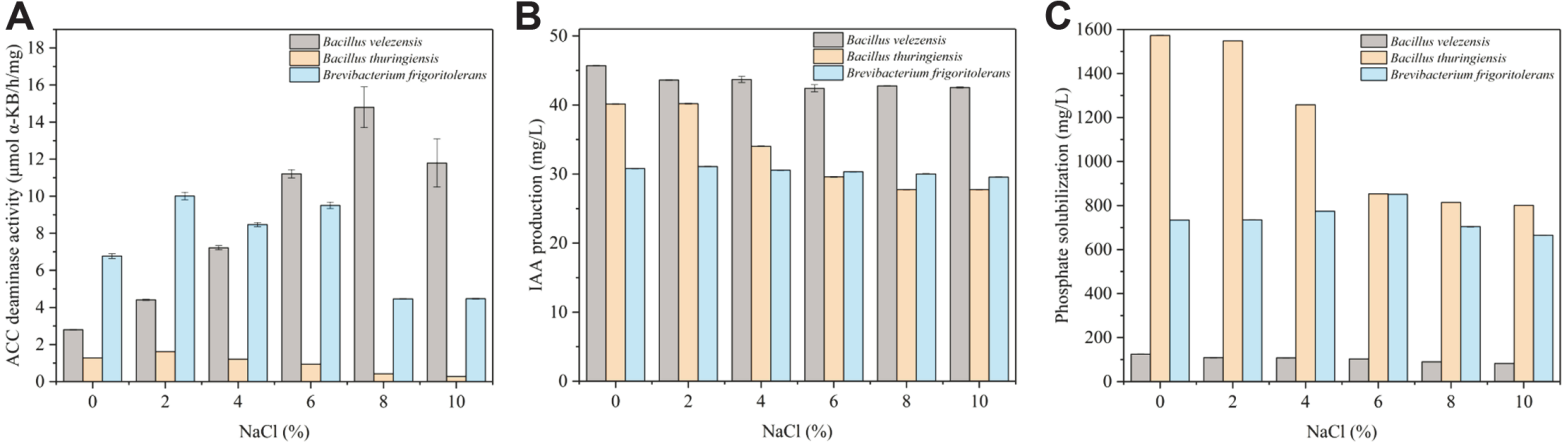
Isolate performance of promoting plant growth under different salinity conditions (0, 2, 4, 6, 8, 10%). (**A**) ACC deaminase activity, (**B**) IAA production, and (**C**) Phosphate solubilization. Data are shown as a mean ± SE of three parallels. The error bars in Fig. 2B and Fig. 2C were not obvious because of small errors in the measurement.

In the present study, we investigated the plant growth-promoting traits of each strain at six salt stress levels of 0, 2, 4, 6, 8, and 10%. As shown in Fig. 2 and Table 3, it can be seen clearly that *B. frigoritolerans* exhibits relatively complete plant growth-promoting traits in the presence of salt stress. The finding is considered significant as it opens up the possibility that the plant growth-promoting traits of the bacterium are taken advantage of to resist salt stress, as shown in Table 4. *B. frigoritolerans* as a novel organism can effectively tolerate high salt stress, but at present, it is widely used to repair contaminated soil [7, 8]. It has also been reported that its drought resistance, inoculated in *Zea mays*, has a certain promotional effect on the number of leaves, and shoot length [32]. However, it is reported that there is still no firm conclusion that *B. frigoritolerans* possesses relatively complete plant growth-promoting traits under abiotic stress, especially under salt stress [14, 33]. In this study, we used *B. frigoritolerans* as a salt-tolerant bacteria isolated from the saline–alkali soil to research its plant growth-promoting traits under salt stress. Further study of the growth promotion activity of the *B. frigoritolerans* under salt stress will make its biological characteristics even more significant. Currently, it is known that the three strains hold the potential to complement each other in constituting a systematic mechanism of growth promotion. That is to say, the combined inoculation of *B. frigoritolerans*, *B. velezensis*, and *B. thuringiensis* on plants will benefit their growth.

### Quantitative Analysis of ACC Deaminase Activity, IAA Production and Phosphate Solubilization

**Fig. 3:**
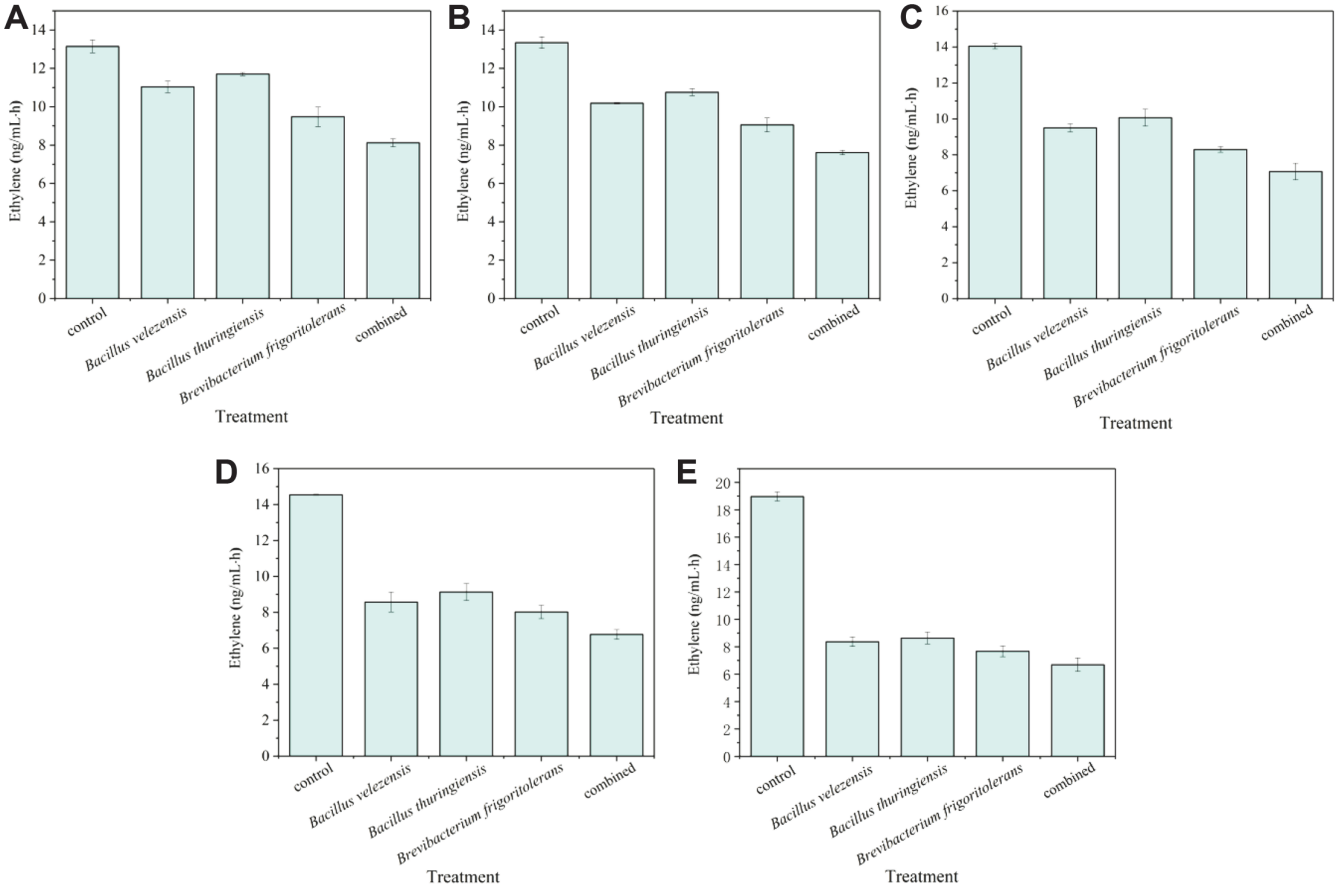
Ethylene content of wheat seeds treated with strains under different NaCl stress. (**A**) 50 mM NaCl, (**B**) 100 mM NaCl, (**C**) 200 mM NaCl, (**D**) 300 mM NaCl, and (**E**) 400 mM NaCl. Data are shown as a mean ± SE of three parallels.

Different NaCl concentrations in the growth medium have different effects on the production of ACC deaminase (Fig. 2A). As shown in Fig. 2A, 2-8% NaCl is effective in inducing the ACC deaminase activity of *B. velezensis*. Besides, the ACC deaminase activity remained at 10.91 μmol α-KB/h/mg even in the presence of 10% NaCl stress. For *B. frigoritolerans*, the trend of ACC deaminase activity was closely related to the growth curve under different salinity conditions. However, salt stress exerted an adverse impact on the activity of deaminase in *B. thuringiensis*. Even so, the screening of isolates in this study showed ACC deaminase activity under salt stress, which broadens the applied biological characteristics. It has been reported that a low level of ACC deaminase activity, above 0.02 μmol α-KB/h/mg, was enough for bacteria to promote plant growth [18]. Apparently, the activity of ACC deaminase produced from the isolates has been shown to actually be higher than the standard. These results indicated that *B. velezensis* and *B. frigoritolerans* can be used as typical salt-tolerant bacteria containing ACC deaminase. Plants exposed to salt stress will produce excessive ethylene, which can severely hinder root development [34]. In a previous study, it was demonstrated that bacteria containing ACC deaminase could contribute to mitigating the negative impact of ethylene stress on plant growth through the conversion of ACC into ammonia and α-ketobutyrate [35]. Therefore, plants with reduced ethylene levels will be able to overcome salt-induced growth inhibition through combination with the ACC deaminase-containing bacteria. For the salinity-stressed wheat seeds, ethylene content was significantly different between untreated and strains-treated wheat seeds (Fig. 3, *p* <0.01). As shown in Fig. 3, at the same level of salinity, the ethylene content of treated wheat seeds was reduced to varying degrees compared with control. In single-strain treatment, the ethylene content of wheat seeds treated with *B. velezensis* or *B. frigoritolerans* was lower in comparison with *B. thuringiensis*. Apparently, wheat seeds treated with the combination of three strains produce the most satisfactory effect in reducing ethylene content.

Fig. 2B shows the IAA production of isolates under different NaCl concentration. In the test of IAA production, the isolates *B. velezensis* and *B. frigoritolerans* were stable to produce IAA under different NaCl concentrations. The IAA of the other strain, *B. thuringiensis*, was decreased with the increase of salt concentration. However, it still had an effective IAA production capacity. Previous study has shown that plant growth-promoting bacteria may help plants resist salt stress by providing the IAA that directly help plants to grow [36]. Other research has shown that the bacteria that were effective in protecting plants from salt stress produce both ACC deaminase and IAA [37]. From this, we can conclude that both *B. velezensis* and *B. frigoritolerans* are potential salt-tolerant bacteria to promote plant growth. According to previous studies, ACC and IAA have significant synergistic effect for promoting plant growth [38, 39]. Synthesized by bacteria, IAA is bound to the roots for developing plants and gets absorbed by plants, thus stimulating the proliferation of plant cells along with endogenous plant IAA [40]. In addition, IAA can convert S-adenosyl methionine into ACC by stimulating the activity of ACC synthase [38]. After being absorbed by bacteria and hydrolyzed to ammonia and α-ketobutyric acid by ACC deaminase, ACC reduced the inhibition of ethylene on root growth for plants. As shown in Fig. 2, *B. thuringiensis* showed positive IAA production while the ACC deaminase activity was indifferent. Therefore, it is necessary to conduct a further research on the synergistic effect of ACC deaminase and IAA activity if *B. thuringiensis* colonizes the plant roots. In general, the IAA levels exhibited by the plant growth-promoting bacteria were positively associated with all indicators of plant growth [41]. Therefore, it can be inferred that the IAA produced by isolates is capable of stimulating primary and lateral root elongation, thus promoting the absorption of nutrients by plant [42]. As shown in Table 4, the root length of wheat inoculated with isolates was greater than control.

The phosphate solubilization of *B. velezensis* and *B. thuringiensis* was decreased with the increase in NaCl stress (Fig. 2C), which is in agreement with the growth activity by themselves (Fig. 1). Even so, among the three strains, *B. thuringiensis* showed the best phosphate solubilization activity. However, the increase of phosphate solubilization activity for *B. frigoritolerans* was consistent with the increase of pH until 6% NaCl. The bacteria showed a 116 mg/l increase in phosphate solubilization in 6% NaCl stress, compared to control. These results indicate that *B. thuringiensis* and *B. frigoritolerans* can release phosphorous from Ca_3_(PO4)_2_, even under high salt stress conditions. The insoluble phosphate can be changed by phosphate-solubilizing microorganisms into available phosphate for plant roots [43]. Apart from the solubilization of phosphorus in Ca_3_(PO4)_2_ by *B. thuringiensis* in this study, another study showed that the Fe–P and Al–P could also be dissolved by *B. thuringiensis* [44]. Therefore, *B. thuringiensis* can be used as a phosphate-solubilization bacteria to provide available phosphate for plants. This consequence was agreed with by Delfim *et al*. [45]. Previous reports have confirmed the phosphate solubilization activity of *B. frigoritolerans* under abiotic stress [14]. This study confirmed that *B. frigoritolerans* retains effective phosphate solubilization in low or high salt conditions, which makes up for the deficiency of previous study. The presence of phosphate-solubilization bacteria provides available phosphate for plant growth via the mechanism of releasing organic acids. Some organic acids can not only reduce the pH value of the soil, but also combine with iron ion, aluminum ion, and calcium ion, so that the insoluble phosphate is dissolved and absorbed by plants [46].

### Qualitative Analysis of Nitrogen Fixation and Siderophore Production

The properties of N_2_-fixation ability and siderophore production of isolated salt-tolerant bacteria strains under salt stress were also essential for promoting plant growth. Table 3 confirmed that *B. thuringiensis* possessed the ability of N_2_-fixation in non-saline or saline conditions. It was reasonable to presume that it can fix the nitrogen elements on the plant roots by a nitrogen fixation mechanism, thereby achieving the purpose of absorbing nutrients. Our conclusion was confirmed by Hongrittipun *et al*. [47]. Nitrogen fixation was mostly found in Rhizobia spp. [48]. In the study, the existence of nitrogen fixation by isolates indicated that free-living bacteria can also fix nitrogen. The conclusion was also agreed upon by Parray *et al*. [49]. Microorganisms can secrete siderophore when small amounts of iron exist in the media. Hence, the qualitative analysis showing siderophore production by a chromazurine blue plate. *B. velezensis* had no siderophore capacity under different levels of salt stress. In contrast, *B. frigoritolerans* has good siderophore production, even under salt stress. *B. frigoritolerans* was previously reported to possess siderophore production under abiotic stress [14]. However, our study further confirmed that *B. frigoritolerans* also has the ability for siderophore production under salt stress. The siderophore can be used as solubilizing agents for iron in minerals or organic compounds under iron-limited conditions [50]. Isolates can secrete an affinity chelator called siderophores to chelate with irons. Iron-siderophores re-enter the cells by means of the interaction of the complexes of special high-affinity cell-surface receptors and iron-siderophore [51].

### Effect of Salt-Tolerant Bacterial Inoculation on the Growth of Wheat Under Salt Stress

**Fig. 4:**
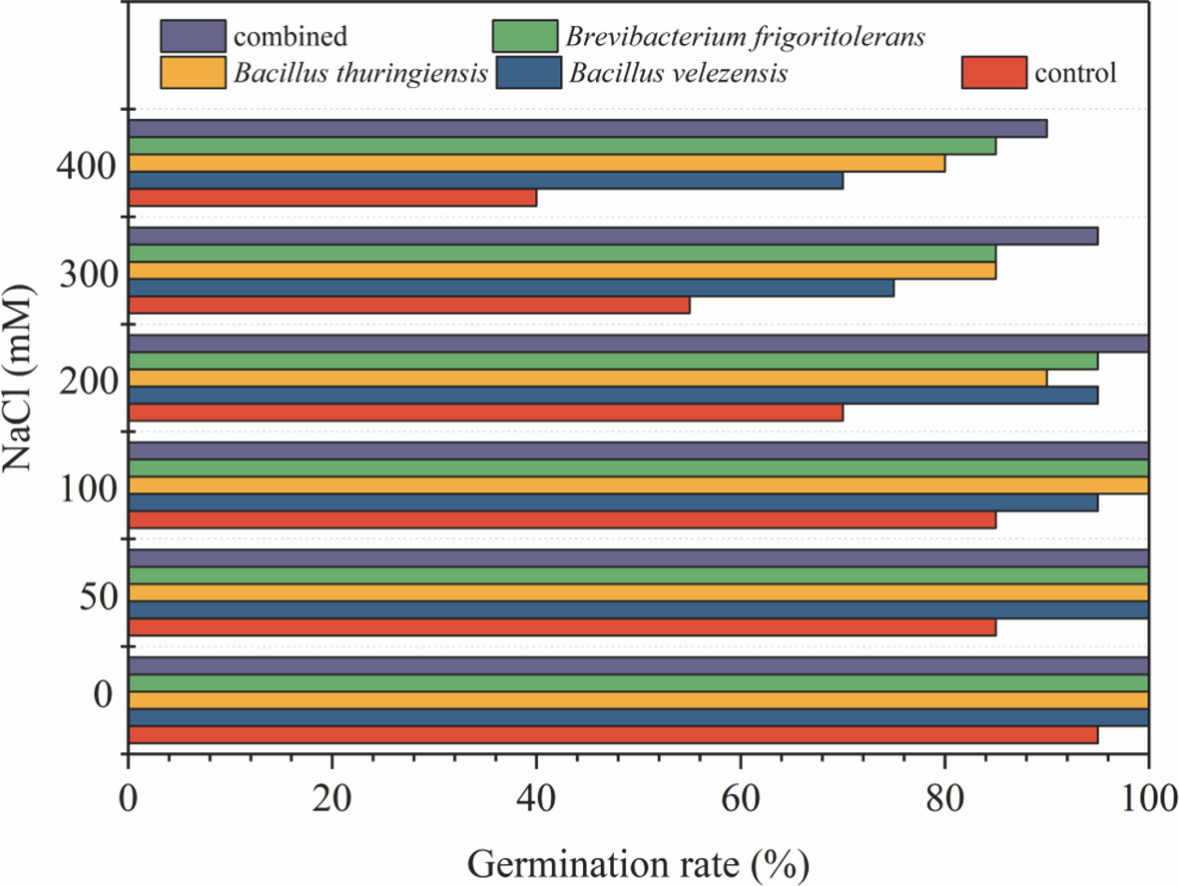
Germination rate of wheat seeds inoculated with strains.

With the increase of salt concentration, wheat seedlings became damaged. The inoculation of a single salt-tolerant microorganism promoted the growth of wheat under salt stress [52]. According to our preliminary experiments shown in Fig. 4, the germination rate was considerably higher when comparing single-strain treatment wheat seeds to inoculation with three strains. For the salinity-stressed wheat seeds, the germination rate was significantly different between untreated and strain-treated wheat seeds. (Fig. 4, *p* < 0.01). Therefore, the strains were treated singly and in combination to alleviate the inhibitory effect of salt stress on wheat growth.

**Fig. 5:**
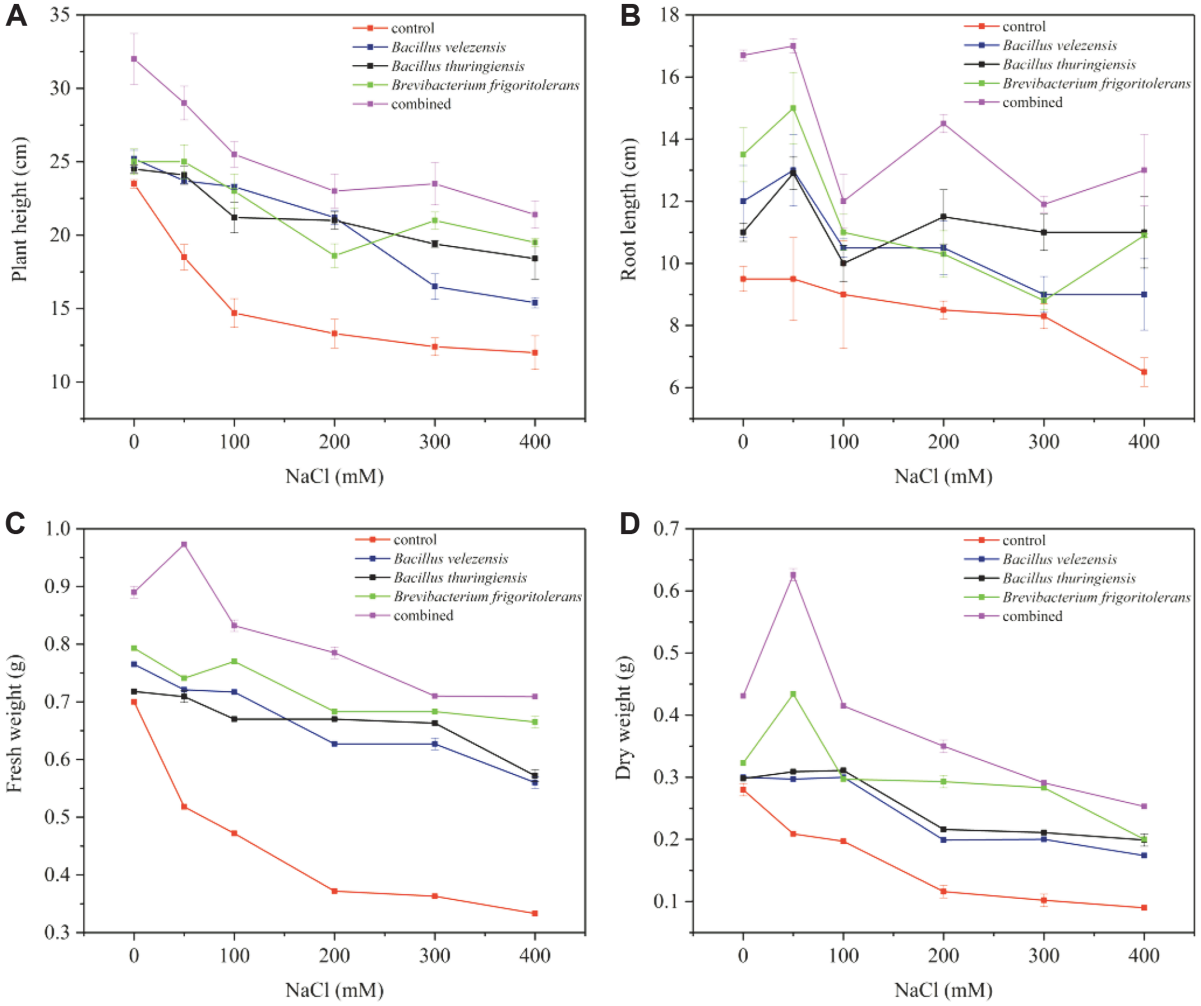
Influence of salt-tolerant bacteria on (A) plant height, (B) shoot length, (C) fresh weight, and (D) dry weight of wheat in the presence and absence of salt stress. Data are shown as a mean ± SE of three parallels.

Wheat inoculated with isolates *B. velezensis*, *B. thuringiensis*, and *B. frigoritolerans*, singly or in combination, showed varying degrees of improvement in plant growth. Fig. 5 and Table 4 show the results of the effects of salt-tolerant bacteria inoculants on wheat plant growth parameters. Fig. 5 clearly shows that the wheat that was uninoculated showed decreased plant parameters with the increase of salt stress. For inoculation alone, *B. frigoritolerans* bettered other strains in terms of the promotion of wheat parameters under salt stress. In addition, we carried out a co-inoculation study on wheat by pot experiments. The results showed the wheat parameters of co-inoculation to be better than the singular, regardless of the presence of salt stress or not; and it was significantly improved, compared to the uninoculated (Table 4). It is worth noting that the effect of treatment was co-inoculation > single > control (Fig. 5). The plant height, root length, and fresh and dry weight showed a (1.78, 2, 2.13, and 2.81)-fold increase in wheat under high salt stress (400 mM), as compared to the control parameters. Compared with single inoculation, plants can obtain better growth conditions from co-inoculation.

The results of pot experiments prove *B. frigoritolerans* to be an effective strain to alleviate the salt stress on plants. The plant growth-promoting traits could be summarized as the strain having a relatively complete system of growth promotion, so it became the most effective single strain inoculation. For co-inoculation, *B. thuringiensis* makes up for the shortfalls in the nitrogen-fixing ability of *B. frigoritolerans* under high salt stress. It also enhanced the ability of phosphate solubilization in the system, while *B. velezensis* enhanced the ACC deaminase activity and IAA production. All three strains were compatible with each other to form a system mechanism of growth promotion. Not only can a strain provide nutrients for another strain, it also creates a favorable conditions [53]. Each strain provides the required nutrients to form a complementary nutrient for plants. Thereby, the growth of different parts in plants was more significantly promoted, so that the integral plant showed a favorable growth condition to enhance the plant resistance against abiotic stress. The result of the pot experiment confirmed the effectiveness of these isolates as salt-tolerant bacteria. Salt-tolerant bacteria promote plant growth by direct or indirect interactions [39]. On the one hand, salt-tolerant bacteria can enhance the ability of plants to withstand salt stress. The bacterium has a special cell membrane and cell wall structure, which can effectively prevent Na^+^ from entering the cell, and maintain a low salt concentration in the cell. Thus, it can play a greater role in promoting growth and the cultivation of salt-tolerant plants. On the other hand, the production of ACC deaminase by salt-tolerant microorganisms can reduce the excess ethylene produced by plants under salt stress. Its extracellular enzymes can decompose or activate the minerals in the soil and enhance the host decomposition of organic matter and the absorption of minerals [54], so as to meet the root requirements for absorbing nitrogen, phosphorus, and potassium, thereby promoting the growth and development of plants. The root absorption area was enlarged when the salt-tolerant microorganisms resided in the roots, and the activity of microorganisms greatly shortened the distance to absorb nutrients [55]. Therefore, the utilization of soil nutrient factors by plant roots was improved. In this study, we demonstrated the effectiveness of *B. frigoritolerans* as a salt-tolerant bacteria to mitigate salt stress, and so enhance plant growth. We also verified the reliability of our idea of the co-inoculation as a biological inoculant to resist salt stress.

## Supplemental Materials



Supplementary data for this paper are available on-line only at http://jmb.or.kr.

## Figures and Tables

**Table 1:** Analysis of the soil characteristics in four different regions of Shandong, China.

Regions	Coordinates	Saltiness (%)	Water content (%)	Volatile solid (%)
Qingdao	(36°28’N, 120°14’E)	0.7	31.75	28.21
Dongying	(37°76’N, 118°98’E)	1.31	20.12	19.71
Jinan	(37°43’N, 117°32’E)	0.33	17.07	17.14
Binzhou	(37°73’N, 117°31’E)	0.45	20.16	18.25

**Table 2:** Identification and taxonomy information of isolates.

Isolate number	Isolated strains showed homology with the bacterial species in the GenBank database^a^				Identified bacterial isolate		
		
	Bacterial species	GB accession number	identity (%)		Strain allocated	GB accession number	Genus
A-1	*Bacillus velezensis* strain CBMB205	NR 116240.1	99.72		*Bacillus velezensis* strain A-1	MW165773	*Bacillus*
A-2	*Bacillus proteolyticus* strain MCCC 1A00365	NR 157735.1	100.00		*Bacillus proteolyticus* strain A-2	MW173214	*Bacillus*
A-3	*Bacillus albus* strain MCCC 1A02146	NR 157729.1	99.86		*Bacillus albus* strain A-3	MW173215	*Bacillus*
A-4	*Bacillus cereus* strain ATCC 14579	NR 074540.1	100		*Bacillus cereus* strain A-4	MW173216	*Bacillus*
A-5	*Bacillus thuringiensis* strain ATCC 10792	NR 114581.1	99.93		*Bacillus thuringiensis* strain A-5	MW165774	*Bacillus*
A-6	*Bacillus wiedmannii* strain FSL W8-0169	NR 152692.1	99.93		*Bacillus wiedmannii* strain A-6	MW173217	*Bacillus*
M-1	*Brevibacterium frigoritolerans* strain DSM 8801	NR 117474.1	100.00		*Brevibacterium frigoritolerans* strain M-1	MW165775	*Brevibacterium*
M-2	*Bacillus amyloliquefaciens* strain NBRC 15535	NR 041455.1	99.72		*Bacillus amyloliquefaciens* strain M-2	MW173218	*Bacillus*

Note: Target strains A-1, A-5, and M-1 were selected for the subsequent experiments.

^a^ Sequence homology was analyzed by blasting the nucleotide sequence homology of the bacterial species in the GenBank database.

**Table 3:** Qualitative analysis of isolates in promoting plant growth performance under different salinity (0, 2, 4, 6, 8, and 10%) conditions.

NaCl (%)												

Isolate	Nitrogen fixation						Siderophore					
	
	0	2	4	6	8	10	0	2	4	6	8	10
A-1	+	+	-	-	-	-	-	-	-	-	-	-
A-5	+	+	+	+	+	+	+	+	+	+	-	-
M-1	+	+	+	+	-	-	+	+	+	+	+	+

Note: + = positive, - = negative.

**Table 4:** The effect of isolates on wheat growth under different salt stress.

NaCl (mM)	Treatment	Plant height (cm)	Root length (cm)	Fresh weight (g)	Dry weight (g)
0	Control	23.5 ± 0.29^c^	9.5 ± 0.40^e^	0.700 ± 0.00^d^	0.280 ± 0.01^d^
	*Bacillus velezensis*	25.2 ± 0.58^b^	12.0 ± 1.15^c^	0.765 ± 0.00^c^	0.300 ± 0.00^c^
	*Bacillus thuringiensis*	24.5 ± 0.29^b^	11.0 ± 0.29^d^	0.718 ± 0.00^d^	0.298 ± 0.00^c^
	*Brevibacterium frigoritolerans*	25.0 ± 0.87^b^	13.5 ± 0.87^b^	0.793 ± 0.00^b^	0.323 ± 0.00^b^
	Combined	32.0 ± 1.73^a^	16.7 ± 0.17^a^	0.890 ± 0.01^a^	0.431 ± 0.00^a^
50	Control	18.5 ± 0.87^d^	9.5 ± 1.33^d^	0.518 ± 0.00^d^	0.209 ± 0.00^d^
	*B. velezensis*	23.7 ± 0.17^c^	13.0 ± 1.15^c^	0.721 ± 0.00^c^	0.297 ± 0.00^c^
	*B. thuringiensis*	24.1 ± 0.63^c^	12.9 ± 0.52^c^	0.709 ± 0.01^c^	0.309 ± 0.00^c^
	*B. frigoritolerans*	25.0 ± 1.15^b^	15.0 ± 1.15^b^	0.741 ± 0.00^b^	0.434 ± 0.00^b^
	Combined	29.0 ± 1.15^a^	17.0 ± 0.23^a^	0.973 ± 0.00^a^	0.626 ± 0.01^a^
100	Control	14.7 ± 0.98^d^	9.0 ± 1.73^d^	0.472 ± 0.00^e^	0.197 ± 0.00^d^
	*B. velezensis*	23.3 ± 0.17^b^	10.5 ± 0.29^bc^	0.717 ± 0.00^c^	0.300 ± 0.00^bc^
	*B. thuringiensis*	21.2 ± 1.04^c^	10.0 ± 0.58^c^	0.670 ± 0.00^d^	0.311 ± 0.00^b^
	*B. frigoritolerans*	23.0 ± 1.15^b^	11.0 ± 0.58^b^	0.770 ± 0.00^b^	0.297 ± 0.00^c^
	Combined	25.5 ± 0.87^a^	12.0 ± 0.87^a^	0.832 ± 0.01^a^	0.415 ± 0.00^a^
200	Control	13.3 ± 0.98^d^	8.5 ± 0.29^d^	0.372 ± 0.00^d^	0.116 ± 0.01^d^
	*B. velezensis*	21.2 ± 0.46^b^	10.5 ± 0.87^c^	0.627 ± 0.00^c^	0.199 ± 0.00^c^
	*B. thuringiensis*	21.0 ± 0.58^b^	11.5 ± 0.87^b^	0.670 ± 0.00^b^	0.216 ± 0.00^c^
	*B. frigoritolerans*	18.6 ± 0.81^c^	10.3 ± 0.75^c^	0.683 ± 0.00^b^	0.293 ± 0.01^b^
	Combined	23.0 ± 1.15^a^	14.5 ± 0.29^a^	0.785 ± 0.01^a^	0.350 ± 0.01^a^
300	Control	12.4 ± 0.60^e^	8.3 ± 0.40^d^	0.363 ± 0.00^e^	0.102 ± 0.01^c^
	*B. velezensis*	16.5 ± 0.87^d^	9.0 ± 0.58^c^	0.627 ± 0.01^d^	0.200 ± 0.00^b^
	*B. thuringiensis*	19.4 ± 0.23^c^	11.0 ± 0.58^b^	0.663 ± 0.00^c^	0.211 ± 0.00^b^
	*B. frigoritolerans*	21.0 ± 0.58^b^	8.8 ± 0.29^c^	0.683 ± 0.00^b^	0.283 ± 0.00^a^
	Combined	23.5 ± 1.44^a^	11.9 ± 0.26^a^	0.710 ± 0.00^a^	0.291 ± 0.00^a^
400	Control	12.0 ± 1.15^e^	6.5 ± 0.46^d^	0.333 ± 0.00^d^	0.090 ± 0.00^d^
	*B. velezensis*	15.4 ± 0.35^d^	9.0 ± 1.15^c^	0.560 ± 0.01^c^	0.174 ± 0.00^c^
	*B. thuringiensis*	18.4 ± 1.39^c^	11.0 ± 1.15^b^	0.572 ± 0.01^c^	0.199 ± 0.01^b^
	*B. frigoritolerans*	19.5 ± 0.29^b^	10.9 ± 0.06^b^	0.665 ± 0.01^b^	0.200 ± 0.00^b^
	Combined	21.4 ± 0.92^a^	13.0 ± 1.15^a^	0.709 ± 0.00^a^	0.253 ± 0.00^a^

The values are mean ± standard errors of 3 replicates, and different letters in each variable denote significant difference (*p* < 0.05) by least significant difference.

## References

[1] Raheem A, Ali B (2015). Halotolerant rhizobacteria: Beneficial plant metabolites and growth enhancement of *Triticum aestivum* L. in salt amended soils. Arch Agron Soil Sci..

[2] Sharma A, Singh P, Kumar S, Kashyap PL, Srivastava AK, Chakdar H (2015). Deciphering diversity of salt-tolerant *Bacilli* from saline soils of Eastern Indo-gangetic plains of India. Geomicrobiol J..

[3] Manninen M, Sandholm TM (1994). Methods for the detection of *Pseudomonas* siderophores. J. Microbiol. Methods.

[4] Masood S, Khan A, Sirajuddin, Zhao X, Javed MT, Khan KS (2016). *Bacillus pumilus* enhances tolerance in rice (*Oryza sativa* L.) to combined stresses of NaCl and high boron due to limited uptake of Na^+^. Environ. Exp. Bot..

[5] Nautiyal CS, Srivastava S, Chauhan PS, Seem K, Mishra A, Sopory SK (2013). Plant growth-promoting bacteria *Bacillus amyloliquefaciens* NBRISN13 modulates gene expression profile of leaf and rhizosphere community in rice during salt stress. Plant Physiol. Biochem..

[6] Vimal SR, Gupta J, Singh JS (2018). Effect of salt tolerant *Bacillus* sp. and *Pseudomonas* sp. on wheat (*Triticum aestivum* L.) growth under soil salinity: A comparative study. Microbiol. Res..

[7] Jariyal M, Gupta VK, Mandal K, Jindal V (2015). *Brevibacterium frigoritolerans* as a novel organism for the bioremediation of phorate. Bull. Environ. Contam. Toxicol..

[8] Tong X, Yuan L, Luo L, Yin X (2014). Characterization of a selenium-tolerant rhizosphere strain from a novel Se-hyperaccumulating plant *Cardamine hupingshanesis*. Scientific WorldJournal..

[9] Kasai K, Mori N, Nakamura C (1998). Changes in the respiratory pathways during germination and early seedling growth of common wheat under normal and NaCI-stressed conditions. Cereal Res. Commun..

[10] Wang S, Feng Q, Zhou Y, Mao X, Chen Y, Xu H (2017). Dynamic changes in water and salinity in saline-alkali soils after simulated irrigation and leaching. PLoS One..

[11] Jha Y, Subramanian RB (2013). Paddy plants inoculated with PGPR show better growth physiology and nutrient content under saline conditions. Chil J. Agr. Res..

[12] Meng Q, Jiang H, Hao J (2016). Effects of *Bacillus velezensis* strain BAC03 in promoting plant growth. Biol. Control..

[13] Gopalakrishnan S, Humayun P, Kiran BK, Kannan IGK, Vidya MS, Deepthi K (2011). Evaluation of bacteria isolated from rice rhizosphere for biological control of charcoal rot of sorghum caused by *Macrophomina phaseolina* (Tassi) Goid. World J. Microbiol. Biotechnol..

[14] Meena, Tara N, Saharan BS (2017). Plant growth promoting traits shown by bacteria *Brevibacterium frigrotolerans* SMA23 Isolated from Aloe vera rhizosphere. Agric. Sci. Digest..

[15] Zhang C, Li XL, Yin LF, Liu C, Zou HW, Wu ZY (2019). Analysis of the complete genome sequence of *Brevibacterium frigoritolerans* ZB201705 isolated from drought- and salt-stressed rhizosphere soil of maize. Ann. Microbiol..

[16] Feng Q, Song YC, Bae BU (2016). Influence of applied voltage on the performance of bioelectrochemical anaerobic digestion of sewage sludge and planktonic microbial communities at ambient temperature. Bioresour. Technol..

[17] Yang J, Yang S (2017). Comparative analysis of *Corynebacterium glutamicum* genomes: a new perspective for the industrial production of amino acids. BMC Genomics.

[18] Penrose DM, Glick BR (2003). Methods for isolating and characterizing ACC deaminase-containing plant growth-promoting rhizobacteria. Physiol. Plant..

[19] Gordon SA, Weber RP (1951). Colorimetric estimation of indoleacetic acid. Plant Physiol..

[20] Prakash J, Arora NK (2019). Phosphate-solubilizing *Bacillus* sp. enhances growth, phosphorus uptake and oil yield of *Mentha arvensis* L. 3 Biotech..

[21] Fiske CH, Subbarow Y (1925). The colorimetric determination of phosphorus. J. Biol. Chem..

[22] Greaves JE, Greaves JD (1930). The microflora of leached alkali soil. Bot. Gaz..

[23] Mudhulkar R, Rajapitamahuni S, Srivastava S, Bharadwaj SVV, Boricha VP, Mishra S (2018). Identification of a new siderophore acinetoamonabactin produced by a salt-tolerant bacterium *Acinetobacter* soli. ChemistrySelect..

[24] Anderson JA, Peters DC (1994). Ethylene production from wheat seedlings infested with biotypes of *schizaphis graminum* (Homoptera: aphididae). Environ. Entomol..

[25] Manivel G, Raj DML, Prathiviraj R, Senthilraja P (2020). Distribution of phylogenetic proximity upon species-rich marine ascomycetes with reference to pichavaram mangrove soil sediment of southern India. Gene Rep..

[26] Ye M, Tang X, Yang R, Zhang H, Li F, Tao F (2018). Characteristics and application of a novel species of *Bacillus*: *Bacillus velezensis*. ACS Chem. Biol..

[27] Jariyal M, Gupta VK, Mandal K, Jindal V, Banta G, Singh B (2014). Isolation and characterization of novel phorate-degrading bacterial species from agricultural soil. Environ. Sci. Pollut. Res..

[28] Yallapragada VVB, Gowda U, Wong D, O'Faolain L, Tangney M, Devarapu GCR (2019). ODX: A fitness tracker-based device for continuous bacterial growth monitoring. Anal. Chem..

[29] Biesta-Peters EG, Reij MW, Joosten H, Gorris LGM, Zwietering MH (2010). Comparison of two optical-density-based methods and a plate count method for estimation of growth parameters of *Bacillus cereus*. Appl. Environ. Microbiol..

[30] González-Pérez CJ, Tanori-Cordova J, Aispuro-Hernández E, Vargas-Arispuro I, Martínez-Téllez MA (2019). Morphometric parameters of foodborne related-pathogens estimated by transmission electron microscopy and their relation to optical density and colony forming units. J. Microbiol. Methods.

[31] Masmoudi F, Abdelmalek N, Tounsi S, Dunlap CA, Trigui M (2019). Abiotic stress resistance, plant growth promotion and antifungal potential of halotolerant bacteria from a Tunisian solar saltern. Microbiol. Res..

[32] Raza FA, Amin A, Faisal M (2015). Desiccation-tolerant rhizobacteria from cholistan desert, Pakistan, and their impact on *Zea mays* L. Pol. J. Environ. Stud..

[33] Tiryaki D, Aydın İ, Atıcı Ö (2019). Psychrotolerant bacteria isolated from the leaf apoplast of cold-adapted wild plants improve the cold resistance of bean (*Phaseolus vulgaris* L.) under low temperature. Cryobiology.

[34] Mahajan S, Tuteja N (2005). Cold, salinity and drought stresses: An overview. Arch. Biochem. Biophys..

[35] Glick BR (2003). Phytoremediation: synergistic use of plants and bacteria to clean up the environment. Biotechnol. Adv..

[36] Qin Y, Druzhinina IS, Pan X, Yuan Z (2016). Microbially mediated plant salt tolerance and microbiome-based solutions for saline agriculture. Biotechnol. Adv..

[37] Glick BR, Cheng Z, Czarny J, Duan J (2007). Promotion of plant growth by ACC deaminase-producing soil bacteria. Eur. J. Plant Pathol..

[38] Glick BR, Penrose DM, Li J (1998). A model for the lowering of plant ethylene concentrations by plant growth-promoting bacteria. J. Theor. Biol..

[39] Glick BR (2012). Plant growth-promoting bacteria: mechanisms and applications. Scientifica (Cairo).

[40] Kende H (1993). Ethylene biosynthesis. Plant Mol.Biol..

[41] Marques APGC, Pires C, Moreira H, António O.S.S. Rangel, Castro PML (2010). Assessment of the plant growth promotion abilities of six bacterial isolates using *Zea* mays as indicator plant. Soil Biol. Biochem..

[42] Xie H, Pasternak JJ, Glick BR (1996). Isolation and characterization of mutants of the plant growth-promoting rhizobacterium *Pseudomonas putida* GR 12-2 that overproduce indoleacetic acid. Curr. Microbiol..

[43] Malboobi MA, Behbahani M, Madani H, Owlia P, Deljou A, Yakhchali B (2009). Performance evaluation of potent phosphate solubilizing bacteria in potato rhizosphere. World J. Microbiol. Biotechnol..

[44] Wang T, Liu M, Li H (2014). Inoculation of phosphate-solubilizing bacteria *Bacillus thuringiensis* B1 increases available phosphorus and growth of peanut in acidic soil. Soil Plant Sci..

[45] Delfim J, Schoebitz M, Paulino L, Hirzel J, Zagal E (2018). Phosphorus availability in wheat, in volcanic soils inoculated with phosphate-solubilizing *Bacillus thuringiensis*. Sustainability..

[46] Zaidi A, Khan MS, Ahemad M, Oves M (2009). Plant growth promotion by phosphate solubilizing bacteria. Acta Microbiol. Immunol. Hung..

[47] Hongrittipun P, Youpensuk S, Rerkasem B (2014). Screening of nitrogen fixing endophytic bacteria in *Oryza sativa* L. J. Agric. Sci..

[48] Duhan JS, Dudeja SS, Khurana AL (1998). Siderophore production in relation to N_2_ fixation and iron uptake in Pigeon Pea-*Rhizobium* Symbiosis. Folia Microbiol..

[49] Parray JA, Jan S, Kamili AN, Qadri RA, Egamberdieva D, Ahmad P (2016). Current perspectives on plant growth-promoting rhizobacteria. J. Plant Growth Regul..

[50] Indiragandhi P, Anandham R, Madhaiyan M, Sa TM (2008). Characterization of plant growth-promoting traits of bacteria isolated from larval guts of diamondback moth *Plutella xylostella* (lepidoptera: plutellidae). Curr. Microbiol..

[51] Sandy M, Butler A (2009). Microbial iron acquisition: marine and terrestrial siderophores. Chem. Rev..

[52] Ansari FA, Ahmad I, Pichtel J (2019). Growth stimulation and alleviation of salinity stress to wheat by the biofilm forming *Bacillus pumilus* strain FAB10. Appl. Soil. Ecol..

[53] Sindhu SS, Gupta SK, Dadarwal KR (1999). Antagonistic effect of *Pseudomonas* spp. on pathogenic fungi and enhancement of growth of green gram (*Vigna radiata*). Biol. Fertil. Soils..

[54] Gongora CE, Broadway RM (2002). Plant growth and development influenced by transgenic insertion of bacterial chitinolytic enzymes. Mol. Breed..

[55] Badri DV, Weir TL, van der Lelie D, Vivanco JM (2009). Rhizosphere chemical dialogues: plant-microbe interactions. Curr. Opin. Biotechnol..

