# Supplementary material for: Biological Inoculant of Salt-Tolerant Bacteria for Plant Growth Stimulation under Different Saline Soil Conditions
Source: J Microbiol Biotechnol. 2020 Dec 25;31(3):398–407. doi: 10.4014/jmb.2009.09032 (PMC9705901; doi:10.4014/jmb.2009.09032)
Supplement: Supplementary file 1 [file jmb-31-3-398-supple.pdf]

https://doi.org/10.4014/jmb.2009.09032

Supplementary Tables

Table 1S. Two-way ANOVA on the ethylene content of wheat seeds treated with strains under different NaCl stress.

	Sum of squares	Mean square	*P*-value
NaCl	13.576	3.394	<0.01
Isolates	497.082	124.270	< 0.01
NaCl * Isolates	99.280	6.205	< 0.01

Table 2S. Two-way ANOVA on the germination rate of wheat seeds treated with strains under different NaCl stress.

	Sum of squares	Mean square	*P*-value
NaCl	8554.089	1710.818	<0.01
Isolates	7444.400	1861.100	< 0.01
NaCl * Isolates	2848.133	142.407	< 0.01

Supplementary Figures

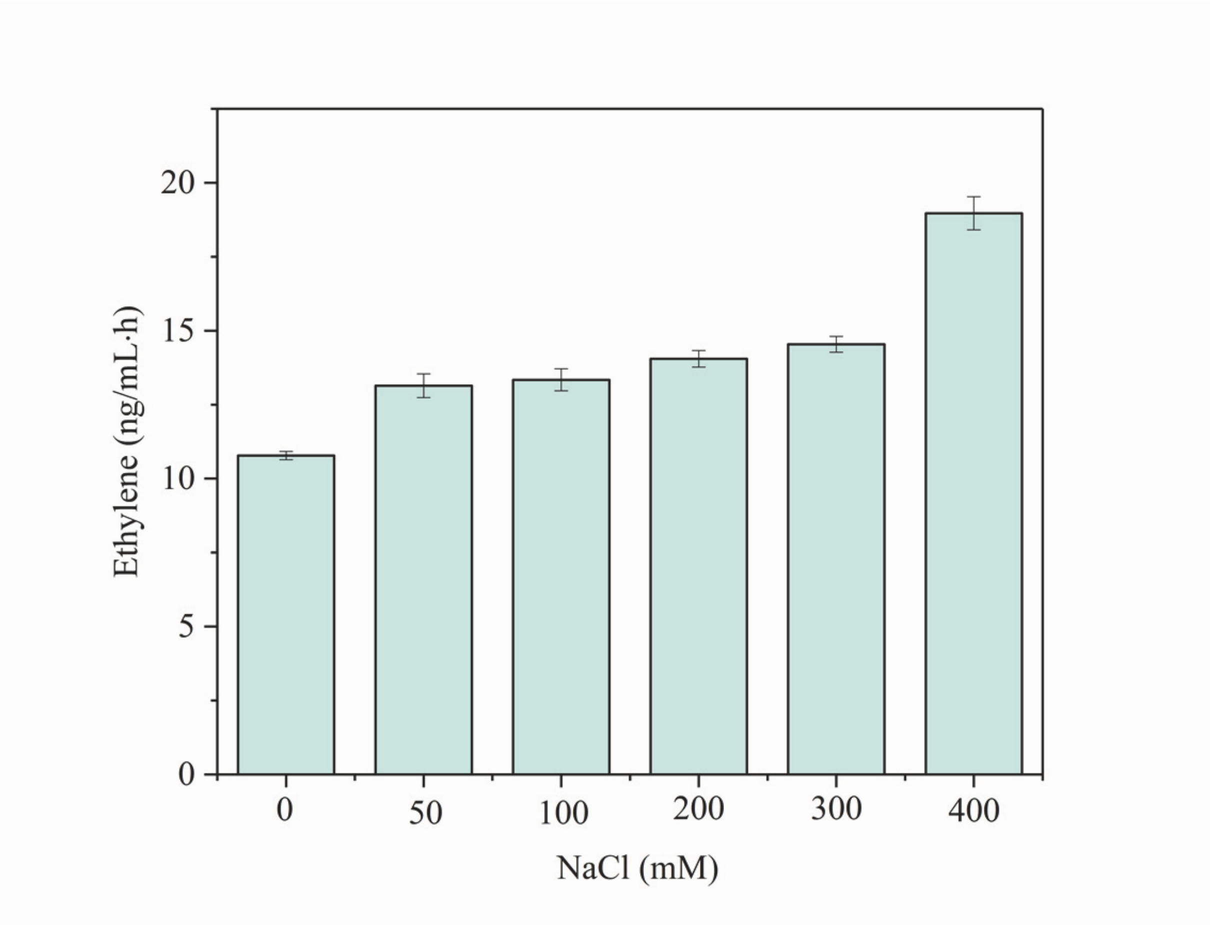

Fig. 1S. Ethylene content in wheat seeds under different salt stress.

Date are shown as a mean ± SE of three parallels.

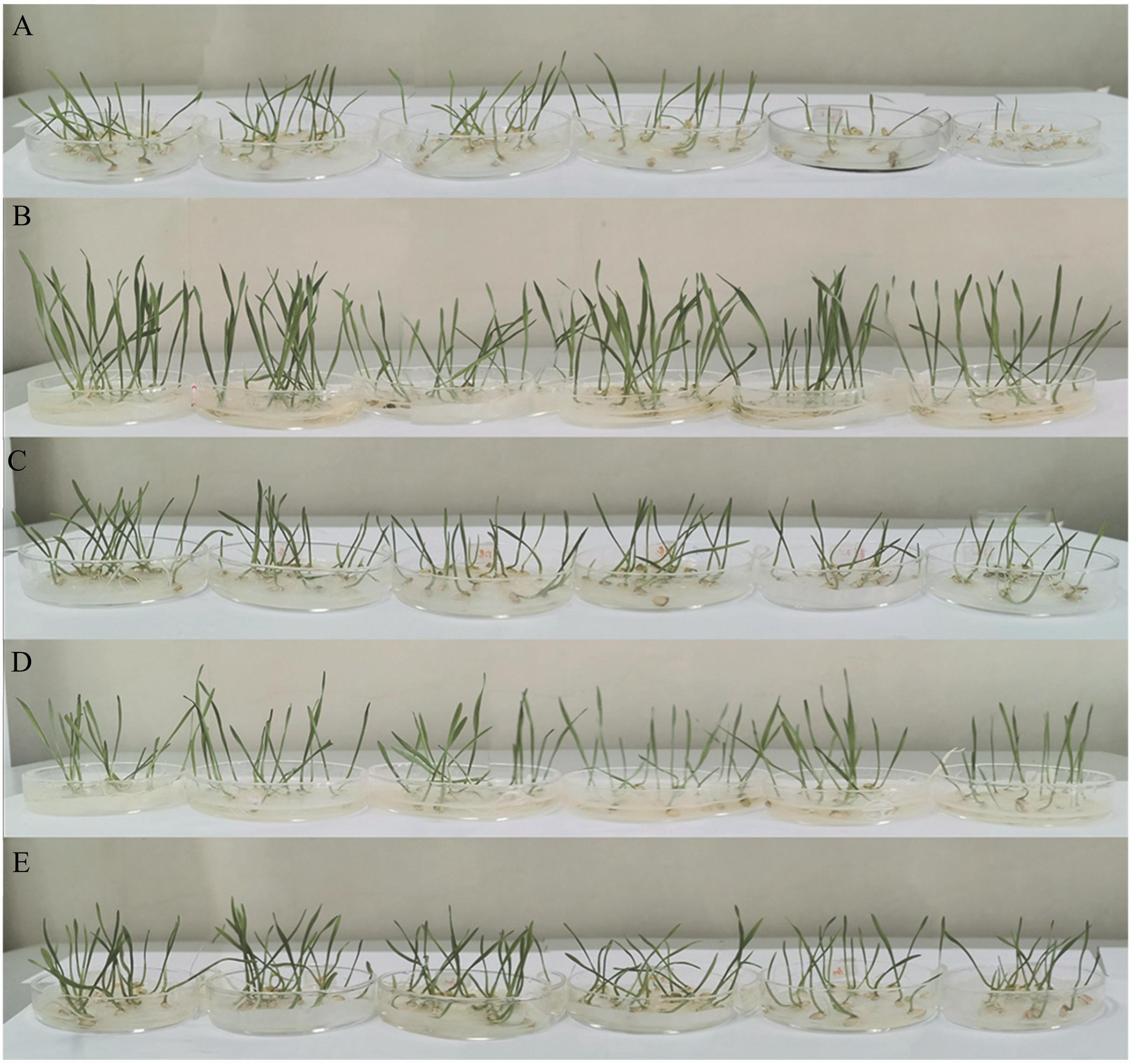

Fig. 2S. Germination of wheat seeds inoculated with strains. (A) control, (B) combined, (C) *Bacillus velezensis*, (D) *Bacillus thuringiensis*, and (E) *Brevibacterium frigoritolerans*.

The NaCl stress of the wheat in every picture from left to right is 0, 50, 100, 200, 300, 400 mM, respectively. Wheat seeds were soaked in bacterial suspensions for 2 h before being moved to a petri dish lined with sterile filter paper to observe their germination rate, with each dish containing 20 wheat seeds. The wheat seeds soaked in sterile broth were treated as a control.

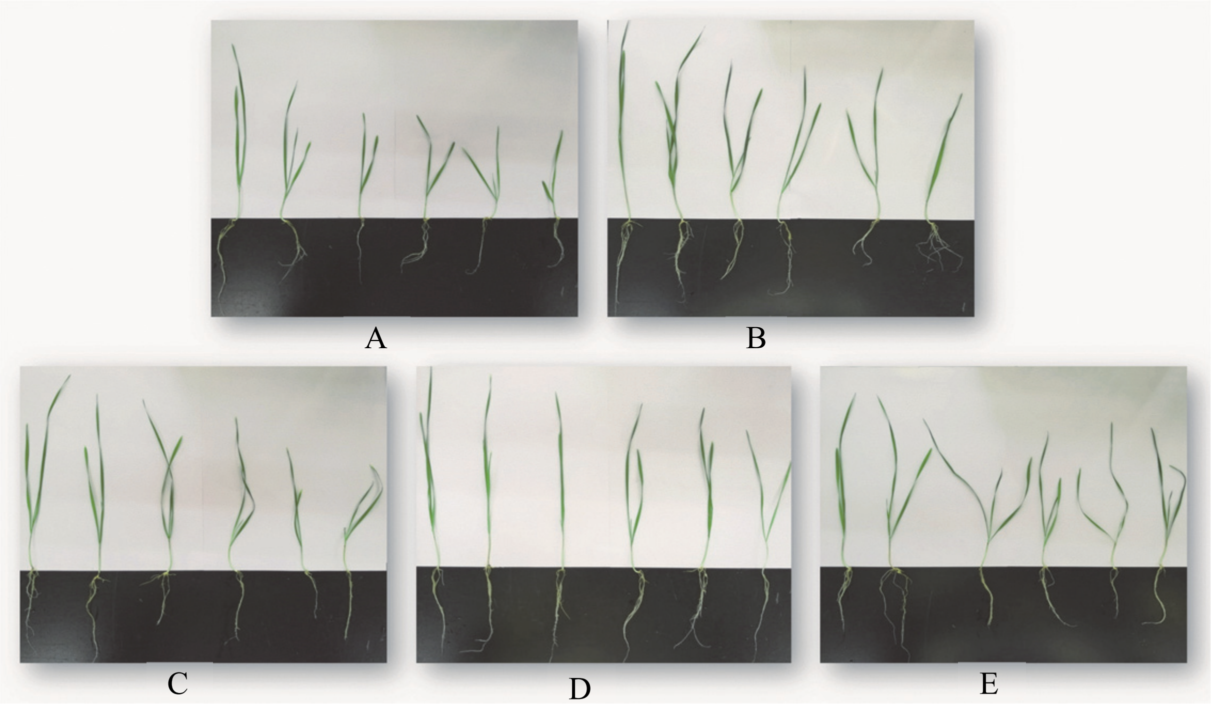

Fig. 3S. Effect of bacterization on the growth of wheat in salt-treated soil. (A) control, (B) combined, (C) *Bacillus velezensis*, (D) *Bacillus thuringiensis*, and (E) *Brevibacterium frigoritolerans*.

The NaCl stress of the wheat in every picture from left to right is 0, 50, 100, 200, 300, 400 mM. The wheat was planted in salt-treated soil. Bacterial suspensions were inoculated weekly on wheat plants in the greenhouse, sterile broth served as a control. One month after treatment, the wheats were removed from the pot to measure their growth parameters.
